# An Analysis of the Time Required for the Diagnosis of ASD and the Factors Influencing Its Duration in a Sample of the Pediatric Population from Poland

**DOI:** 10.3390/jcm13206255

**Published:** 2024-10-20

**Authors:** Krzysztof M. Wilczyński, Lena Cichoń, Aleksandra Stasik, Katarzyna Kania, Natalia Rodak, Michał Wizner, Małgorzata Janas-Kozik

**Affiliations:** 1 Department of Psychiatry and Developmental Age Psychotherapy, Medical University of Silesia in Katowice, 40-055 Katowice, Poland; 2 John Paul II Child and Family Health Center in Sosnowiec, 41-218 Sosnowiec, Poland; 3 Students’ Scientific Club, Department of Psychiatry and Developmental Age Psychotherapy, Medical University of Silesia in Katowice, 40-055 Katowice, Poland

**Keywords:** ASD, child, Poland, diagnosis

## Abstract

**Background/Objectives**: Early diagnosis of autism spectrum disorder (ASD) is a very important factor for improving the quality of life of people on the spectrum, but it still remains a major problem in Europe, especially concerning girls. In this study, we tried to answer the question of what factors affect the age of diagnosis in Poland. Additionally, we tried to establish the time between the first visit to the mental health center (MHC) and the diagnosis in this population, and what factors affect its length. **Methods**: 77 children were randomly recruited among the patients who came to local MHC at the Child and Family Health Centre in Sosnowiec. All participants were tested using the ADOS-2 protocol. In addition, the study used the TAS-20 test and BDI. **Results**: The mean age of the first enrollment in the MHC was 9.09 years for girls and 6.42 for the boys. The time needed to obtain a diagnosis from the first visit was consecutively 2.90 years and 4.29 years, but the difference was not statistically significant (*p* > 0.05). Obtaining a different psychiatric diagnosis did not affect the average time to diagnosis and the age of diagnosis. **Conclusions**: The results of the study indicate that the diagnosis of ASD is still a significant problem both in Poland and in the world. Obtaining an accurate diagnosis requires significant time, and it frequently involves consulting multiple specialists. The diagnostic process should be flexible, and the specialist should always take into account the axial symptoms but remain aware that the “overdiagnosis” of ASD can also have harmful consequences for the child.

## 1. Introduction

Autism spectrum disorder (ASD) includes a heterogeneous group of nosological entities that are characterized by difficulties in establishing and maintaining social communication and social interactions, as well as limited, inflexible, repetitive patterns of behavior, interests, and activities [1]. Current studies from the literature indicates that individuals who are eligible for a diagnosis according to the DSM-5/ICD-10 criteria represent a few percent of the population, but the exact demographics are extremely variable. In Europe, they range from 0.5 to 3%, but, for example, in Japan, they reach up to 9% of the population [2,3,4,5]. Such large differences in epidemiology are on one hand probably due to sociodemographic factors resulting from the mutual determinants of the course of evolution of a given society with the cultural conditions shaped by it in the “particular” rather than the “universal” sense, as defined by Vogeley and Roepstorff in a 2009 publication [6]. On the other hand, this may be partly related to the continuous development of diagnostic criteria and the large differences between, for example, the American DSM and the ICD used in Europe [7]. In both classifications, the concept of ASD has undergone significant modifications over the years, from a rigid dichotomous division into autistic and non-autistic people to a gradual liberalization of this view. However, Lorna Wing has already suggested that the actual nature of ASD may be based on the concept of the so-called autistic continuum, i.e., the hypothesis that everyone actually presents a certain level of “autisticness” but only some to a level that could require support and justify a diagnosis. The latest diagnostic criteria (DSM-5 and ICD-11) reflect this evolution. According to the most recent classification that is coming into use in Europe (ICD-11), axial symptoms of ASD include the following [8]:

1. Maintaining social interaction and communication;
2. Tendencies to restricted, inflexible, repetitive patterns of behavior, activities, and interests that are clearly unusual or excessive for the individual.

Early diagnosis and appropriate therapeutic interventions have a significant impact on the quality of life and functioning of people on the spectrum. Delaying the diagnosis often exposes such a person to years of peer difficulties and significant stress, which may lead to the development of other psychiatric disorders and illnesses, such as depressive episodes, in the future [9]. In the analyses from the Center for Disease Control and Prevention (CDC) [10], as well as in most international guidelines, it was indicated that although ASD is suspected even as early as 18 months of age, a reliable diagnosis is possible after the age of 24 months. Despite this, the early diagnosis and implementation of appropriate interventions are still a major problem in Europe, especially in the context of girls [11]. Many clinicians often believe that ASD cannot be diagnosed, e.g., before the age of 5, or that symptom improvement following appropriate therapeutic interventions rules out the diagnosis. As a result, numerous international organizations are working to establish guidelines for ASD diagnosis, with a strong emphasis on early detection of the disorder.

In Europe, the leading guidelines are by the National Institute for Health and Care Excellence (NICE), published in 2011 and updated in 2017 and 2021, respectively [12]. In these guidelines, the authors point to the need to create diagnostic teams including the following:

(1) Pediatrician and/or child and adolescent psychiatrist;
(2) Speech therapist;
(3) A clinical psychologist with education and experience in working with people with ASD.

People under the age of 3 should be referred to this type of team, especially if they have regressed speech or social competencies. If a regression of speech or motor skills is observed after the age of 3, a neurological and pediatric consultation should be performed first to rule out other disorders. The NICE guidelines also highlight that no diagnostic tools specifically designed for ASD should be used as the sole basis for either confirming or excluding a diagnosis. These tools are merely supplementary and intended to assist the diagnostician, ideally a doctor, such as a child and adolescent psychiatrist. These guidelines also list the most common, definitely erroneous grounds for excluding ASD: (1) proper eye contact, facial expressions directed to family members, (2) current history of proper pretend play, proper speech development, (3) improvement of observed difficulties after appropriate therapeutic interventions, and (4) previous examinations excluding the diagnosis of autism spectrum disorders despite new data,, e.g., from the interview. They also point to factors that may hinder diagnosis, requiring caution in the diagnostic process. These factors include (1) less than 24 months of age, (2) no early childhood history, (3) adolescence, (4) complex cases involving other psychiatric disorders (e.g., ADHD), sensory deficits (e.g., blindness or deafness), or motor disorders (e.g., cerebral palsy).

The 2014 guidelines of the American Academy of Child and Adolescent Psychiatry (AACAP) [13] emphasize three points in their recommendations for the diagnosis of ASD.

1. The AACAP recommends that screening for ASD should include all children as part of the assessment of psychophysical development and, if necessary, as part of psychiatric consultations, already in infancy and in young children. Screening should be carried out primarily within the primary care clinic using standard questionnaires (e.g., ASRS or AQ).
2. In the case of positive results of screening or referral for diagnostics by a doctor/psychologist or other medical professionals., a full mental state examination should be carried out along with an interview by a psychiatrist, taking into account the DSM-5 diagnostic criteria. In its recommendations, the AACAP emphasizes that while many diagnostic tools with varying sensitivity and specificity have been developed, they should never replace comprehensive diagnostics and are meant to serve only a supportive role.
3. All children diagnosed with ASD should undergo a full medical diagnosis without exceptions, including a hearing test, tuberous sclerosis tests, and a genetic test including karyotype analysis, tests for fragile X and, in justified cases, an aCGH microarray.

Finally, it is also worth mentioning the standards published in Poland: ”Organizational standards and substantive standards for the second and third reference level centers: Mental Health Center for Children and Adolescents/Psychiatric Outpatient Clinic for Children and Adolescents/Psychiatric Therapeutic and Rehabilitation Day Wards for Children and Adolescents and Highly Specialized Round-the-Clock Psychiatric Care Centers/Admission Rooms/Psychiatric Day Wards Diagnostic and Therapeutic for Children and Adolescents” edited by Prof. Anita Bryńska, Prof. Tomasz Wolańczyk, and Prof. Agnieszka Słopień. In the chapter on guidelines for dealing with people on the spectrum, a standard of conduct for the diagnosis of ASD can be found. Just like in the international guidelines, it is emphasized that the diagnosis of ASD can be made at any age—at the earliest, even at 18–24 months of age. The authors point out that in younger children, the diagnosis may be subject to a greater error. It is recommended that various professionals be involved in the diagnosis process, including child and adolescent psychiatrists, psychologists, educators, and/or neurologopedists. According to the standard, the diagnostic process should include an interview with the parent/caregiver, observation of the diagnosed person, tests to assess cognitive functions, observation/obtaining an interview from various environments (e.g., school), assessment of the intellectual level and finally providing information about the diagnosis to parents/guardians.

Therefore, according to international guidelines, the diagnosis of ASD should be made as early as possible, preferably around the age of 24 months, based primarily on a psychiatric examination and analysis of the developmental history—preferably within multidisciplinary teams—and diagnostic tools should only play a role supporting the diagnostic process.

Late delayed diagnosis is a common problem around the world. Makino et al. [14] in their 2021 literature review summarized publications on the diagnosis of ASD in different countries and on different continents and showed that the average period from the first visit to diagnosis ranged from 12 to 55 months.

To the best knowledge of the authors, at the time of the preparation of this study, there were no papers available in the literature analyzing either the time of diagnosis of ASD or the factors affecting its duration in the Polish population. For this reason, it was of interest to evaluate how long it takes to obtain a diagnosis of ASD in Poland and what factors affect this procedure.

## 2. Materials and Methods

### 2.1. Study Participants

The participants were recruited from among patients of outpatient MHC at the Department of Psychiatry and Psychoterapy of Developmental Age (OKPiPWR) at the John Paul II Child and Family Health Center in Sosnowiec Sp.zo.o. (Sosnowiec, Poland). Informed consent to participate in the study was obtained after information was provided from both parents and participants.

Inclusion criteria included (1) confirmation of a diagnosis of autism spectrum disorder previously made by a specialist in child and adolescent psychiatry using the ADOS-2 protocol and (2) age between 12 and 19 years. The exclusion criteria from the study and control groups included (1) concomitant intellectual disability, (2) epilepsy, (3) known genetic, neurometabolic, etc., background of the observed symptoms (e.g., fragile X syndrome), autism plus, (4) poor somatic state—severe acute somatic diseases affecting cognitive functions/decompensation of chronic diseases, diagnosis of serious liver, kidney or heart disease, unstable hypothyroidism. In the control group, an additional exclusion criterion was having people with a family history of autism spectrum disorders.

According to the inclusion criteria, all participants were within intellectual norm—the mean IQ value measured by the BINET scale was IQ = 98.74 (95% CI: 95.8–101.62; SD: 12) and showed no statistically significant differences between the sexes (*p* > 0.05). The mean BINET IQ was 98.72 (95% CI: 93.77–103.63) among girls and 98.6 (95% CI: 94.8–102.3) among boys.

### 2.2. Psychometric Analysis

All participants were tested using the ADOS-2 protocol with the use of modules corresponding to the age and language level of the participants. Further analyses focused primarily on the total score (ADOS:WC), the social affect domain (SA), and the domain of repetitive and stereotyped patterns of behavior and interests (RRB).

In addition, the study used the following:

(a) The Toronto Alexithymia Scale–20 (TAS-20) tests three dimensions: difficulty in identifying feelings, difficulty in describing feelings, and outward-oriented thinking. Items are rated by the respondent on a 5-point Likert scale. The score range is between 20 and 100 points, with higher scores indicating a higher severity of alexithymia. The total alexithymia score is the sum of the responses in all 20 items, while the score for each factor of the subscale is the sum of the responses in that subscale. The Polish language version of the scale was validated, for which the Cronbach α coefficient is 0.73 for the overall score; 0.55 for the “difficulty in describing emotions” subscale; 0.71 for the “difficulty identifying emotions” subscale; and 0.51 for the “outward-oriented thinking style” scale [15,16].
(b) Beck Depression Scale (BDI)—In the present study, the BDI scale was used to exclude depressive symptoms in patients enrolled in the study due to the tendency of the TAS-20 scale to overestimate the results in people with depression indicated in the literature. Individuals scoring above 10 points would be excluded from further analysis. In the initial cohort selected for the study, no participant exceeded this threshold.

### 2.3. Ethics Committee Approval

The study was carried out with the consent of the SUM Bioethics Committee issued by Resolution No. KNW/0022/KB1/123/18/19 of 8 January 2019.

### 2.4. Statistical Analysis

The statistical analysis was performed using StatSoft Statistica software version 13. The distributions of the studied variables were analyzed and verified using the Shapiro–Wilk test so that they remain consistent with the normal distribution. The adopted level of statistical significance for Spearman’s rank correlation analyses was *p* = 0.05. The Bonferroni correction for multiple comparisons has been abandoned in most cases due to the nature of the analyzed correlations and comparative analyses, where the existence of relationships is justified on the basis of existing literature data. Lastly, a multiple regression analysis was conducted to examine the interactions among the variables under investigation.

## 3. Results

The data collected as part of the study were pseudonymized. The study was conducted among 77 children. The demographic data and outcome of selected questionnaires utilized in the study are presented in Table 1. Additionally, information was gathered from interviews and documentation regarding the date of the initial visit to the MHC at local or other facilities, as well as the date of the ASD diagnosis. These data were utilized to calculate the average time required to obtain a diagnosis from the first visit, with the results also presented in Table 1. In regard to the cut-off point for clinically significant diagnosis of alexithymia; it was exceeded by 23 subjects (29.8%). Among girls, there were 7 (22.5%), and among boys, there were 16 (34.7%).

There was no statistically significant correlation between the age of the first visit to MHC and the overall length of diagnosis (r = 0.05; *p* = 0.74).

There was no statistically significant correlation between the level of intellect measured by the BINET scale and the time needed for diagnosis and age at the beginning of the diagnostic process (rho = 0.13; *p* > 0.05). A statistically significant correlation was not found between age at the start of diagnosis and the duration of diagnosis (rho = 0.08; *p* > 0.05).

Finally, an analysis of the total outcome of ADOS-2 test (total and comparative score), its subscales and TAS-20 between the sexes was also performed. Statistically significant differences were obtained in the total result of ADOS-2 as well as both subscales—the results are presented in Table 2.

The prevalence of aggressive behavior as a possible predictor of the time of diagnosis was also analyzed. In the described group, their occurrence was present in 47.4% of the population (n = 37), and they differed statistically significantly depending on sex in the χ^2^ test (F: 32.2% vs. M: 58.7%; *p* = 0.021). In terms of the age of diagnosis, no statistically significant differences were observed between those presenting and not presenting aggressive behaviors. Such statistically significant differences were observed for the age of onset of diagnosis and the time of diagnosis, where individuals who presented aggressive behaviors, started the diagnostic process earlier (10.89 (95% CI: 9.70–12.08) vs. 11.87 (95% CI: 10.5–13.24); *p* = 0.046) but required more time to make a correct diagnosis (4.51 (95% CI: 3.46–5.55) vs. 2.98 (95% CI: 2.24–3.73) *p* = 0.044).

Finally, the first diagnoses received by the patients during the diagnostic process—before they were diagnosed with ASD—were analyzed. In the analyzed group, 77.9% (n = 60) of patients had another diagnosis prior to ASD. Interestingly, the acquisition of a different psychiatric diagnosis did not impact the average time taken to receive an ASD diagnosis or the age at which the ASD diagnosis was made. There were also no differences in the range of these parameters between specific diagnostic categories. The summary is presented in Table 3.

An analysis of the correlation between the questionnaires and the age of the first visit to the MHC and the time that passed between the first visit and the diagnosis of ASD was also conducted. The age of the first visit to MHC was correlated only with the total score of the ADOS-2 study (rho = -0.32; *p* = 0.0092) and the ADOS-2 social affect subscale (rho = -0.29; *p* = 0.012). Meanwhile, the time it took for a specialist to diagnose ASD depended on the TAS-20 score (rho = −0.30; *p* = 0.0049).

On the basis of the results obtained, it was also decided to conduct a multiple regression analysis in order to create a model for predicting the time needed to make a diagnosis of ASD based on the results of the analyzed questionnaires and the ADOS-2 study. The model was constructed using the stepwise regression method and obtained statistical significance with *p* = 0.0004. The ADOS-2 total and TAS-20 test results were included in the analysis. The parameters of the obtained model are presented in Table 4.

## 4. Discussion

In the presented analysis, attention is drawn primarily to the very late onset of the diagnostic process and its extension in time, which ultimately translated into the late age of the ASD diagnosis itself. Such a postponement of the correct diagnosis is very disadvantageous for patients, leading to a situation in which appropriate therapeutic interventions are often implemented only in the teenage years. This, in turn, according to the available literature, translates into a narrower range of available and actual interventions undertaken by patients, and as a result, their limited effectiveness in the later years of life or the motivation of the patient himself [17]. People who received this type of late diagnosis indicate that from their perspective it was related to the loss of therapy opportunities and improvement of their functioning [18]. Moreover, a delay in diagnosis is associated with a higher risk of developing other psychiatric disorders (m.in. mood disorders) and numerous difficulties in both private and professional life. Of course, despite this, the very fact of receiving a diagnosis, even at such a late age, was a breakthrough moment for most of the respondents—especially girls—which was associated with a significant improvement in the quality of life [9,11,18].

In accordance with the preliminary hypotheses, the age of initiation of the diagnostic process was significantly higher in girls than in boys. Clinical practice suggests that this phenomenon is partially attributable to the tendency of girls to seek help at the MHC due to co-occurring disorders, such as depressive episodes that frequently arise from the challenges associated with undiagnosed ASD, particularly during their teenage years. In addition, the ability to mask one’s symptoms, usually quite good social functioning, and fewer externalizing behaviors mean that girls are generally diagnosed less frequently and later than boys, which is a phenomenon often indicated in the literature [9,19]. The obtained results confirm these assumptions, indicating that both the total result of ADOS-2 obtained in the examination and the frequency of aggressive behaviors were lower among girls than boys. On the other hand, the occurrence of aggressive behaviors translated into an earlier age of diagnosis and, interestingly at the same time extended the time of diagnosis. The latter observation may be due to the fact that diagnosticians, focusing on co-occurring disorders that generate aggressive behavior, initially do not notice the features of ASD. Secondly, it should be noted that despite the fact that the difference in diagnosis time between the sexes was not statistically significant, there is a clear tendency towards shorter diagnosis times among girls in the studied population. Therefore, if a higher percentage of boys is observed in the group of people with aggressive behaviors, it will naturally lead to a longer time of diagnosis in this population. An interesting question is the direction of this relationship—whether it is the occurrence of aggressive behaviors, to which boys are more prone (in the present study), that actually prolongs the time needed to make a diagnosis, or whether the dominance of boys in the group of people presenting aggressive behaviors causes a longer time of diagnosis—which would theoretically result from the clinical picture of ASD in boys. Considering the greater severity of ASD traits, along with supporting literature [19,20], the first hypothesis appears significantly more plausible. In the context of the time of the diagnosis itself, it should be noted that it was unusually long and stretched over years. This is a problem not only for the situation in Poland but also for other countries. This is indicated, among others, by the aforementioned literature review by Makino et al. [14], where the period from the first visit to diagnosis ranged from 12 to 55 months. Parents usually pointed out that the diagnosis process was in fact a “pointlessly protracted struggle for this diagnosis”, and that there were numerous delays and obstacles in the way—including the fact that the analyzed papers indicated that it was usually only the third or even subsequent specialists who made the correct diagnosis. Moreover, most of the papers indicated that the diagnosis process was described by parents as “overly complicated and unclear.” The data obtained in the present study paint a picture analogous to the one presented in the review by Makino et al. However, it should be remembered that this is data obtained from other countries and populations. Unfortunately, it is impossible to compare the results obtained with studies on other Polish populations. This is due to the fact that, according to the authors’ knowledge, at the time of writing this paper, the presented analysis is the first one concerning the situation in Poland.

An interesting question is, what really affects the time of diagnosis? Most of the available studies focus on the distinction between people with a very high level of ASD features and low levels of functioning and high-functioning people with low levels of ASD features. Speech development disorders, characteristic symptoms of stereotypical behaviors (hand-waving and tiptoe-walking), or increased social deficits are very good predictors of a short diagnosis time [21]. The problem begins when we move on to discuss the differences in the timing of diagnosis among high-functioning children with ASD. Interestingly, in the present study—conducted on a group of such people—the severity of autistic traits measured in the ADOS-2 study was not statistically significant at the time of diagnosis. In the logistic regression analysis, the severity of alexithymia, as measured by the TAS-20 scale, emerged as a statistically significant, albeit weak, predictor. Alexithymia is a multifaceted construct, defined as difficulties in understanding or identifying one’s own feelings, as well as describing them in the context of relationships with other people [15,22]. Its exact nosological position remains unclear due to the fact that some authors perceive alexithymia as a symptom of other disorders (e.g., personality), and some as an independent nosological entity—which is related to the fact that diagnostic criteria are proposed in the literature. However, there is no unambiguous, coherent definition of this construct, and it is believed to be based on both psychological and biological factors [23]. The prevalence of alexithymia in the general population (with TAS-20 scores >= 60) is estimated to be approximately 10%, with sex-disaggregated analyses typically showing a prevalence of approximately 9.4% in men and 5.2% in women [24]. In the present study, the incidence of alexithymia in accordance with this criterion was 29.8%; it was several times higher than in studies on the general population. Nevertheless, this result aligns with existing literature, which demonstrates a strong association between alexithymia and ASD. Some studies report that up to 40–65% of patients diagnosed with ASD achieve a very high score on alexithymia tests using the TAS-20 scale [25,26,27]. There are a number of inconsistencies in the findings regarding emotion processing in ASD, and thus new hypotheses suggest that difficulties in this area may be due to concomitant alexithymia. They are supported by publications according to which children with higher severity of alexithymia have a lower level of social competence regardless of the diagnosis of ASD. Although emotional deficits are not the primary diagnostic feature of ASD, difficulty perceiving emotions in others, lack of empathy, and other difficulties in processing emotions are often used as diagnostic markers of ASD. This can also be seen in the present study where the results on the TAS-20 scale were a better predictor of the time of diagnosis than the severity of autistic traits measured by the ADOS-2 scale. This phenomenon may be due to the general difficulty in properly diagnosing ASD. There are numerous publications in the literature on mechanisms that, while not included in the criteria for diagnosis, may be the basis of particular axial symptoms of ASD [28,29,30,31]. They are the basis of some tests used in the diagnosis of ASD and, remaining in the minds of diagnosticians, can distort their expectations of a particular patient, leading to underdiagnosis. An example of this is a disorder in the so-called theory of mind. Deficits in the theory of mind occurring in the course of ASD, leading to characteristic disorders of social cognition, disorders in decoding non-verbal messages (e.g., gestures or facial expressions), and difficulties in reading metaphors, are sometimes associated in the literature with co-occurring alexithymia rather than the strict clinical picture of ASD [32,33]. Moreover, many people with ASD, especially girls, over a certain age are able to solve theory of mind tasks without any problems—either because of the proper functioning of this mechanism or because of the ability to compensate for deficits with the help of the intellect. So, basing a diagnosis of ASD solely on theory of mind tests in such a person, of course, will negate their occurrence—which would be a wrong decision.

Thus, the results of the study indicate that the diagnosis of ASD is still a significant problem both in Poland and in the world. The long time needed to obtain the correct diagnosis, the frequent need to change diagnosticians in order to obtain it, and the lack of understanding of the true nature of the ASD diagnosis by specialists is a common problem. This is most likely due to the individual conceptualization of the case of an “autistic” person by diagnosticians. If a patient who meets the formal criteria for ASD does not fit the “picture” considered by the diagnostician to be “autistic”, he or she will not receive the diagnosis he or she needs to receive appropriate therapeutic support. Taking into account the richness and heterogeneity of ASD symptomatology, it can be assumed that no one is able to create such a concept that would be suitable for all neuroatypical people. This, in turn, indicates that the diagnostic process should be flexible, and the diagnostician should always take into account the axial symptoms for the diagnosis of ASD and the patient’s well-being.

On the other hand, specialists must also be aware that the “overdiagnosis” of ASD (as well as lack of diagnosis) can also have harmful consequences for the child, e.g., through an excessively limited range of social and educational experiences or a long-term impact on the formation of his or her identity. An erroneous diagnosis of ASD may result from irregularities at the level of the diagnostic process, e.g., incorrectly interpreted data or incompetently conducted diagnostic examinations/tests. At the population level, there is also an excessive diagnosis of ASD in children with other neurodevelopmental disorders in order to improve access to various forms of therapy or financial support, as the regulations in force in many countries often provide access to help for children with ASD in an unequal way compared to those with other difficulties (even though they require these experiences just as much as their peers with ASD). According to Fombonne [34], in order to avoid the overdiagnosis of ASD, the diagnostic process must be guided by the following principles: (a) rely on several sources or data sources, rather than just one; (b) supplement the assessment of current functioning with other data confirming the course of autistic symptomatology; (c) demonstrate that the functional impairment is due to underlying autistic disorders and not to co-occurring conditions or specific contextual constraints; (d) provide clinically informed and validated procedures to integrate all measurements at the individual level. Interestingly, these principles seem to be equally helpful not only in preventing overdiagnosis but also in preventing underdiagnosis of ASD [35].

Future research on this topic should primarily focus on expanding the catalog of potentially relevant factors modifying the time of diagnosis. Furthermore, it is necessary to design and verify the effectiveness of programs aimed at improving the situation not only in Poland but also in other countries. Possible impacts should focus not only on quality verification and possible improvement of the training of diagnosticians. It is very important to emphasize the role of preschool and schoolteachers, pedagogues, trainers, and the like in the early identification of people potentially requiring diagnosis. Similarly, this type of intervention should be addressed to pediatricians and nursing staff—these professional groups usually observe children’s development from an early age and should have the competence to identify neuroatypicality early and refer the child for specialist diagnosis.

When interpreting the presented results, the issue of the limitations of the presented study should be taken into account. First, the analysis covers only a certain range of factors that can shape the time of diagnosis—as can be seen, i.e., in the regression model. Factors such as family environment or social support may have an impact on the time to diagnosis, but since they were not controlled for in the article, the reliability of the conclusions is affected. The second issue is the limited size of the analyzed group. Although the size used here theoretically provides sufficient statistical power to reliably draw conclusions about the general population. However, it should always be remembered that the numbers of several dozen people are small and are always a source of potential error in the interpretation of the results.

## Figures and Tables

**Table 1:** Summary of the mean values of the variables analyzed in the presented study.

Mean (95%CI)	Whole Group n = 77	Females n = 31 (40.2%)	Males n = 46 (59.7%)	*p*-Value U Mann-Whit.
Age	14.24 (13.7–14.7)	14.1 (12.9–14.5)	14.4 (13.9–15)	n/s
First visit in MHC (age)	7.5 (6.33–8.66)	9.09 (7.38–10.8)	6.42 (4.88–7.96)	0.002
Time needed for diagnosis (years)	3.7 (3.06–4.34)	2.90 (2.21–3.59)	4.29 (3.32–5.25)	n/s
Age of diagnosis	11.5 (10.5–12.4)	12.3 (11.1–13.2)	9.84 (8.1–11.2)	n/s
TAS-20	53.1 (49.9–56.4)	50.03 (45.66–54.41)	55.83 (51–60.61)	n/s

**Table 2:** Comparison of ADOS-2 (total and comparative scores) and its subscales results between genders. ADOS-2 SA: ADOS-2 social affect; ADOS-2 RRB: ADOS-2 repetitive and stereotyped patterns of behavior and activity; F: females, M: males.

	Total [×(95%CI:)]	F [×(95% CI:)]	M [×(95% CI:)]	*p*-Value
ADOS-2 comp. score	6.89 (6.33–7.45)	5.95 (5.23–6.67)	7.35 (6.61–8.09)	0.002
ADOS-2 total	13.67 (12.05–15.22)	11.9 (10.3–13.4)	15.44 (13.8–17.04)	0.002
ADOS-2 SA	11.79 (10.4–13.5)	10.5 (9.04–11.9)	13.08 (11.76–14.4)	0.004
ADOS-2 RRB	1.88 (1.23–2.54)	1.4 (0.59–2.22)	2.37 (1.88–2.87)	0.016

**Table 3:** Distribution of diagnoses received by patients before an ASD diagnosis was obtained. ^1^ Percent of the total population.

	N (%)	F/M	χ^2^ *p*-Value F/M
F90.0/F90.1	25 (32.4%)	4/20	0.02
F91	14 (18.2%)	2/10	0.04
F34	4 (5.19%)	4/0	-
F32	4 (5.19%)	1/3	-
F4x (F40 and F43)	8 (10.38%)	5/3	-
Other	5 (6.49%)	0/5	-
Overall	60 (100%)	16 (51.6%) ^1^/41 (89.1%) ^1^	

**Table 4:** Multiple regression analysis parameters for a model predicting the time from the first visit to a mental health clinic to the diagnosis of ASD.

	Corrected R^2^	F	*p*-Value
MODEL	0.85	21.63	0.00345
TAS-20	−0.11	13.42	0.014
ADOS-2	-	-	>0.05

## Data Availability

All of the acquired data are included within the study.
